# Using single cell sequencing data to model the evolutionary history of a tumor

**DOI:** 10.1186/1471-2105-15-27

**Published:** 2014-01-24

**Authors:** Kyung In Kim, Richard Simon

**Affiliations:** 1 Biometric Research Branch, National Cancer Institute, 9609 Medical Center Dr., MSC 9735 Bethesda, MD 20892, USA

## Abstract

**Background:**

The introduction of next-generation sequencing (NGS) technology has made it possible to detect genomic alterations within tumor cells on a large scale. However, most applications of NGS show the genetic content of mixtures of cells. Recently developed single cell sequencing technology can identify variation within a single cell. Characterization of multiple samples from a tumor using single cell sequencing can potentially provide information on the evolutionary history of that tumor. This may facilitate understanding how key mutations accumulate and evolve in lineages to form a heterogeneous tumor.

**Results:**

We provide a computational method to infer an evolutionary mutation tree based on single cell sequencing data. Our approach differs from traditional phylogenetic tree approaches in that our mutation tree directly describes temporal order relationships among mutation sites. Our method also accommodates sequencing errors. Furthermore, we provide a method for estimating the proportion of time from the earliest mutation event of the sample to the most recent common ancestor of the sample of cells. Finally, we discuss current limitations on modeling with single cell sequencing data and possible improvements under those limitations.

**Conclusions:**

Inferring the temporal ordering of mutational sites using current single cell sequencing data is a challenge. Our proposed method may help elucidate relationships among key mutations and their role in tumor progression.

## Background

The application of next-generation sequencing technologies has enabled researchers to detect cancer genome alterations on a large scale. However, most current sequencing technologies can only provide the genetic content of cell averages, because the sequencing target is a mixture of many cells in the tumor. Signals obtained from current bulk sequencing technologies only reflect the overall characteristics of a population of sequenced cells, so variation among different cells within a tumor cannot be evaluated. Recently developed single cell sequencing technology can sequence the genome extracted from a single cell. The intra-tumoral heterogeneity of tumors can potentially be observed by sequencing many individual cells within a single tumor.

Single cell sequencing data provide an opportunity for inferring the genealogy of an individual cell. Although cell genealogy is generally not of interest, mutation records of cells can be used to model a tree of the history of the mutations in a tumor [1]. This can serve to identify the earliest mutations that are present in all sub-clones and help us understand how key mutations are accumulated through a clonal evolutionary process that results in a heterogeneous tumor. A major challenge in the model development of these tree is the high error rate of single cell sequencing technology (for example, high allelic dropout ratios; see Hou et al. [2]). Consequently, a computational model of the mutation tree should properly incorporate the uncertainty of the data using a careful statistical model.

Several studies have used single cell sequencing technologies to investigate the genetic heterogeneity of tumors. Navin et al. [3] performed copy number variation analysis on breast tumors using low coverage single nucleus sequencing. The study aimed to cluster tumor subpopulations and reconstruct the clonal evolution of the tumors. They constructed a phylogenetic tree of sample cells and separated tumor subpopulations based on the distances in the tree between the samples. Hou et al. [2] performed mutation analysis using exome sequencing data from 58 single cells of an essential thrombocythemia (ET) tumor. This was the first study to identify candidate mutations related to tumor progression using DNA sequence mutations in individual cells. They tried to establish the monoclonal origin of the ET tumor using population analysis of the single cell sequences. Li et al. [4] performed exome sequencing of 66 single cell samples of a muscle-invasive bladder transitional cell carcinoma to phylogenetically group the samples. Clonal structures and subpopulations of the tumor were proposed using population analysis similar to Hou et al.’s study. All of these studies address the issue of tumor population structure and clonal evolution using single cell sequencing, but they do not address temporal relationship between mutated genes, which is a key and necessary factor to fully understand tumor progression.

Our study differs from those described above, all of which only infer the phylogenetic relationships among the samples. We attempt to infer the evolutionary mutation tree, which indicates the temporal and lineage relationships among DNA sequence mutation sites. The evolutionary mutation tree identifies which mutations occurred in the same lineage, and which occurred in different lineages. We wish to locate individual mutations on the branches of the phylogenetic tree, and thereby identify the temporal and clonal relationships among the mutations. The earliest mutation site is positioned at the root, and the relative distances from the root to other sites in the tree are used to infer the time-frame of the occurrences of the further mutations. To this end, we first propose a new statistical method to determine the mutation order of any two sites using the single cell sequencing data. This model is likelihood-based and accommodates sequencing errors. Based on the pairwise mutation orders of all sites, we then construct a mutation tree using the minimal spanning tree algorithm. We also provide a method to estimate the proportion of time from the earliest mutation event of the tumor to the most recent common ancestor (MRCA) of the cells sequenced.

Gusfield developed a gene tree algorithm to estimate the mutation order of the DNA mutation sites of DNA sequences [5]. The gene tree algorithm in Gusfield’s study assumes that the DNA sequences are sufficiently accurate that the lineages are uniquely determined. Each lineage of the gene tree encodes the path of mutations that occurred in the DNA sequence corresponding to that lineage, under the condition of the perfect phylogeny (the condition that there is a unique tree consistent with the sequences, and the assumption that mutations at sites occur exactly once). Griffiths et al. [6,7] used Gusfield’s algorithm in conjunction with coalescent theory to estimate the ages of the mutations. However, Gusfield’s gene tree is not applicable to single cell next-generation sequencing (NGS) data; the perfect phylogeny condition is violated because of sequencing error. Our approach does not use Gusfield’s gene tree algorithm; rather, it first estimates all pairwise mutation orders based on a coalescent process, and then constructs a DNA mutation site gene tree that corresponds to those mutation orders.

Desper et al. [8] developed an oncogenetic tree algorithm to infer causal relationships among copy number aberrations in a small number of chromosomal regions based on comparative genomic hybridization data. However, the algorithm is not appropriate for the analysis of single cell data because it cannot handle a large number of variables or false positive copy number calls in the dataset.

We apply our algorithm in the Results section to the 18 sites that Hou et al. [2] identified as being important in their single cell sequencing dataset. We estimate the mutation tree of the 18 sites, and the proportion of time to the MRCA of the samples. In the Conclusions and discussion section, we conclude and discuss future directions. In the Methods section, we present a statistical model for inferring pairwise mutation orders. This includes introducing a Bayesian approach for computing the prior and posterior distributions of mutation orders. Furthermore, we describe an algorithm for constructing the minimal spanning mutation tree based on the pairwise mutation orders.

## Results

Our model and analysis of single-cell sequencing data were motivated by Hou et al. [2]. The data were obtained using single cell exome sequencing from a patient with ET. The dataset consists of genotypes from 58 single cell sequencing and two tissue sequencing data (one tumor tissue and one normal tissue), for 712 mutation sites in specific genes. The normal tissue sequencing data were all homozygous, except for missing sites, and were used as the reference wildtype for our analysis. The full dataset is publicly available through the cited article’s journal website.

We analyzed the 18 nonsynonymous mutation sites selected as important by Hou et al. [2]. The authors first selected 171 sites among 712 mutation sites, based on whether those mutations were in the genes’ coding region, and on their likelihood of having a functional gene product. Among the 171 sites, 78 nonsynonymous somatic mutation sites were identified, and then using SIFT algorithm [9] and COSMIC database [10], they further screened the potential list down to the 18 chosen mutation sites. The genes containing the 18 mutation sites in the dataset were ABCB5, ANAPC1, ARHGAP5, ASNS, DLEC1, DMXL1, DNAJC17, FAM115C, FRG1, MLL3, NTRK1, PABPC1, PDE4DIP, RETSAT, SESN2, ST13, TOP1MT, and USP32. The total number of entries in the full data was 41,296 (712×58), but 58% of entries in the dataset did not satisfy the quality criteria of the paper [2], so they were treated as missing. About 45% (468) of the entries were missing for the selected dataset of the 18 mutation sites.

We transformed the genotypes of the mutation sites into integers by counting the number of mutations: 0 to represent wildtype, 1 to represent heterozygous mutations, and 2 to represent homozygous mutations. We excluded all the missing entries in our analysis. Table 1 shows the transformed genotype dataset used in our analysis. It consists of genotypes for the 18 mutation sites and the 58 samples.

**Table 1:** Transformed genotype dataset for the 18 mutation sites and the 58 samples

**Gene (site) ∖**																													
**Sample ID**	**1**	**2**	**3**	**5**	**6**	**7**	**8**	**9**	**12**	**16**	**18**	**19**	**20**	**22**	**24**	**25**	**26**	**29**	**30**	**31**	**36**	**37**	**40**	**41**	**43**	**44**	**45**	**47**	**48**
PDE4DIP (A →G)	-	-	-	1	1	0	-	0	-	-	-	0	-	-	-	-	-	-	1	-	-	-	0	-	0	-	1	0	-
NTRK1 (A →G)	-	0	-	-	1	-	-	-	-	2	-	2	1	1	-	-	1	-	2	-	-	1	-	-	0	0	-	-	2
SESN2 (C →T)	1	1	1	-	-	-	-	1	1	1	2	-	-	1	-	-	-	0	0	1	-	2	2	-	0	2	0	1	1
ARHGAP5 (G →A)	-	1	-	-	-	0	0	2	-	-	-	-	0	1	1	-	-	-	-	0	-	1	-	-	0	-	0	0	-
DNAJC17 (C →G)	1	-	1	-	-	-	-	0	-	1	0	-	-	-	-	-	-	2	1	-	-	-	-	-	1	2	-	2	1
USP32 (C →T)	-	-	-	1	0	-	-	1	-	-	-	-	0	0	0	-	-	-	0	-	-	-	-	-	-	1	0	1	0
ANAPC1 (G →A)	-	-	-	1	0	1	0	1	-	1	0	-	1	1	0	1	1	0	2	-	0	-	-	0	0	0	0	1	-
RETSAT (C →T)	-	0	-	1	0	0	-	-	-	0	-	-	-	0	-	0	0	-	0	-	0	-	0	-	0	-	0	0	-
ST13 (G →A)	0	0	-	-	-	-	0	1	1	1	-	0	1	-	-	1	-	-	1	0	1	1	-	0	0	1	-	1	-
DLEC1 (T →C)	-	-	-	-	-	-	-	1	-	1	-	-	0	2	2	2	-	-	1	-	-	1	-	-	-	-	2	1	1
FRG1 (G →A)	0	0	1	0	0	-	0	-	0	0	0	0	-	-	-	0	-	-	0	-	-	-	0	0	0	-	0	1	0
DMXL1 (G →A)	2	-	-	1	-	0	-	1	-	2	-	-	-	2	-	0	0	1	1	-	2	-	1	-	0	-	-	-	-
FAM115C (T →C)	-	-	-	-	0	-	-	-	-	-	-	0	-	-	-	-	0	-	-	-	-	-	-	-	-	2	0	0	-
MLL3 (C →T)	-	-	0	-	0	0	-	0	0	0	1	-	0	-	0	-	0	0	1	0	1	1	0	-	0	0	0	-	-
ABCB5 (G →T)	1	-	-	1	-	-	1	2	1	0	1	-	-	0	0	-	-	2	2	-	-	2	-	-	-	-	1	-	-
ASNS (T →A)	0	0	0	0	0	0	0	1	-	0	0	0	-	0	0	1	1	0	-	0	1	1	1	-	0	1	0	-	0
PABPC1 (C →T)	0	0	0	0	0	0	0	-	1	-	0	-	-	0	0	0	-	-	0	0	-	0	0	-	1	0	-	0	0
TOP1MT (A →G)	-	-	-	-	-	-	-	-	-	-	-	0	-	-	-	-	-	-	-	0	2	2	1	-	-	1	-	1	-
**Gene (site) ∖**																													
**Sample ID**	**49**	**50**	**52**	**54**	**56**	**60**	**61**	**63**	**66**	**69**	**70**	**72**	**73**	**74**	**76**	**78**	**79**	**80**	**82**	**86**	**87**	**88**	**89**	**90**	**91**	**93**	**94**	**97**	**100**
PDE4DIP (A →G)	-	-	-	-	1	-	0	-	-	-	0	-	0	1	0	0	-	0	0	1	-	0	-	-	-	-	1	1	-
NTRK1 (A →G)	-	2	1	0	-	-	1	-	-	0	1	1	-	0	0	1	1	0	1	-	0	-	-	0	-	0	0	1	2
SESN2 (C →T)	1	0	0	0	1	-	1	0	1	-	-	1	0	1	2	2	-	1	1	1	2	1	0	0	1	2	2	1	1
ARHGAP5 (G →A)	1	0	-	-	0	0	-	-	0	-	1	0	0	0	-	0	0	1	1	-	0	0	-	-	0	1	0	1	0
DNAJC17 (C →G)	1	-	1	0	1	1	-	1	1	1	1	2	-	1	0	1	0	1	1	-	1	-	0	-	-	1	-	2	1
USP32 (C →T)	0	0	1	0	1	0	0	-	1	0	0	-	-	-	-	-	0	-	0	1	-	0	1	1	-	0	0	0	-
ANAPC1 (G →A)	0	0	1	-	0	-	-	0	0	1	-	0	-	0	0	-	-	-	1	0	-	0	0	-	-	-	0	-	0
RETSAT (C →T)	1	0	1	0	-	0	1	0	0	1	0	0	-	0	1	1	-	1	0	0	-	0	0	-	1	1	0	0	-
ST13 (G →A)	-	0	1	-	1	1	1	1	1	1	0	1	-	0	-	0	1	1	0	1	1	1	0	1	1	0	-	1	0
DLEC1 (T →C)	-	-	-	2	-	2	-	-	1	-	-	-	-	2	2	0	1	-	-	-	-	-	-	-	0	2	-	-	1
FRG1 (G →A)	0	0	0	-	0	0	1	1	0	-	0	0	0	0	0	0	0	0	1	1	0	-	1	0	-	0	0	0	0
DMXL1 (G →A)	1	-	1	-	1	1	0	0	1	-	1	-	-	-	-	2	-	-	-	0	-	-	0	-	1	-	0	2	-
FAM115C (T →C)	-	-	-	-	1	-	-	-	-	-	-	-	-	0	-	1	-	-	-	-	0	-	1	-	-	-	-	-	-
MLL3 (C →T)	0	1	1	1	-	1	0	-	0	-	0	-	0	0	-	-	0	0	0	0	0	-	0	0	-	1	-	-	0
ABCB5 (G →T)	-	-	-	-	1	1	1	-	1	0	0	-	-	1	0	-	1	0	-	1	-	-	1	-	-	-	-	0	-
ASNS (T →A)	-	1	-	0	-	1	-	-	0	-	0	0	-	0	0	1	0	1	0	0	0	0	0	0	0	-	-	1	0
PABPC1 (C →T)	0	0	0	0	-	0	-	0	0	0	0	-	-	0	-	0	-	0	1	0	0	-	0	-	0	1	-	0	0
TOP1MT (A →G)	1	2	-	2	2	0	-	-	-	-	1	-	1	-	1	-	-	1	2	-	-	-	-	2	1	-	0	-	1

The first column shows the 18 genes which have the 18 mutation sites and the corresponding point mutations for the sites. The 58 sample ids represent the sample ids used in the paper [2]. 0 represents homozygous wildtype, 1 represents heterozygous mutation and 2 represents homozygous mutation. - represents missing entry. For example, 0, 1 and 2 of the site for SESN2 represent DNA base composition of CC, CT and TT, respectively.

### Mutation tree of the 18 sites

**Figure 1:**
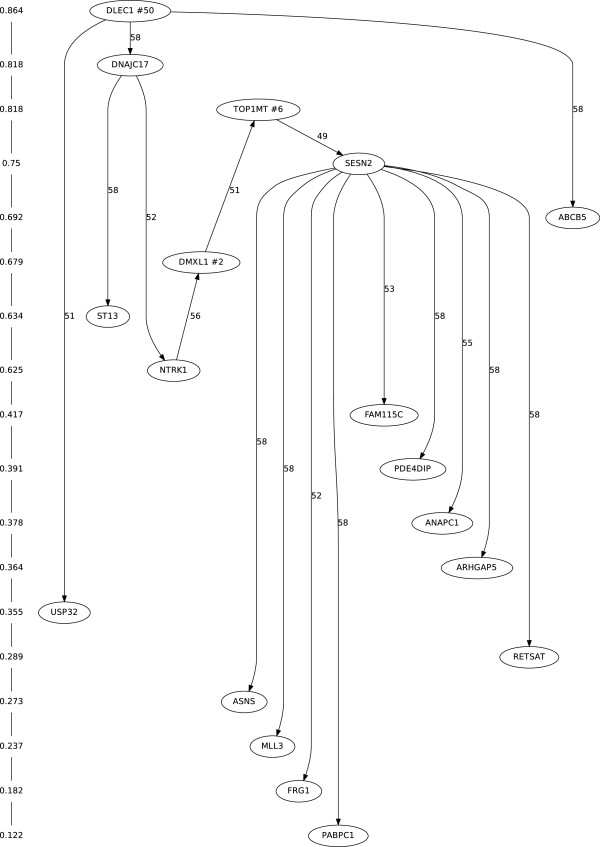
**Mutation tree with the 18 important sites in [**[2]**].** The pairwise mutation order relations of the 18 sites were selected by the Edmonds’ minimal spanning tree algorithm. Directions of branches in the tree represent the mutation orders between the corresponding two sites. The sites of the tree are ordered according to mutation rates which are shown in the left side. DLEC1 has the highest mutation rate 0.864 and PABPC1 has the lowest mutation rate 0.122. The leave-one-out replication result with 58 leave-one-out mutation trees is also summarized in the figure. Each branch has a number which represents how many times the branch is found in the 58 leave-one-out replication. Numbers in the three nodes (DLEC1, DMXL1 and TOP1MT) represent how many times each node is found as root in the 58 leave-one-out replication. The figure was produced by the R software [11], RBGL R package [12], and Graphviz [13].

Figure 1 shows the mutation tree for the genotypes of the 18 important sites in Table 1. This tree describes the mutation orders for the 18 sites, which were selected using the minimal spanning tree algorithm discussed in the Methods section. The tree consists of one root, DLEC1, 12 terminal leaves, and five internal nodes.

Except for two branches, NTRK1 to DMXL1 and DMXL1 to TOP1MT, the directions of the branches for all sites agree with the order of mutation rates in the figure. That is, the tail of the branch corresponds to the site with the higher mutation rate, and the head corresponds to the site with the lower mutation rate. For example, the root node, DLEC1, has the highest mutation rate at 0.864, and PABPC1 has the lowest mutation rate 0.122. Some interesting findings discussed in the original paper [2] are also shown in the tree. SESN2, known for being involved in DNA damage and genetic instability [14], is positioned as the direct ancestor of the nine mutation sites. In Hou et al.’s original study the authors selected four sites (SESN2, ST13, DNAJC17, and TOP1MT) among the 18 sites that have the highest likelihood of being involved with ET initiation and/or progression. Among the four sites, DNAJC17, TOP1MT and SESN2 are aligned sequentially, which may indicate how those sites are related functionally in ET progression. Additionally, NTRK1 is also found in the path of DNAJC17, TOP1MT and SESN2.

The branches in the tree represent partial order relations among sites, but the absence of a branch between two sites does not necessarily mean that the mutations of the two sites occur in different lineages. Because of high sequencing error rates and a large number of missing entries, alternate mutation trees consistent with the pairwise posterior probabilities are also possible. Additional file 1: Table S1 provides posterior probabilities for pairwise order relations for all 18 sites.

We also evaluated the stability of the mutation tree algorithm by a leave-one-out replication. We constructed 58 trees, each with the same sites, but a different sample omitted for each tree. We summarized how frequently the root and the branches in the full sample tree were also found in the 58 trees in Figure 1. For example, the root DLEC1 in the full sample tree was found at the root in 50 of the sub-sample trees, and each branch in the full sample tree was also found in at least 49 of the sub-sample trees. Therefore, we determined that the pairwise orders of the minimal spanning tree were robust using this evaluation setup.

### Time estimation to the MRCA

**Figure 2:**
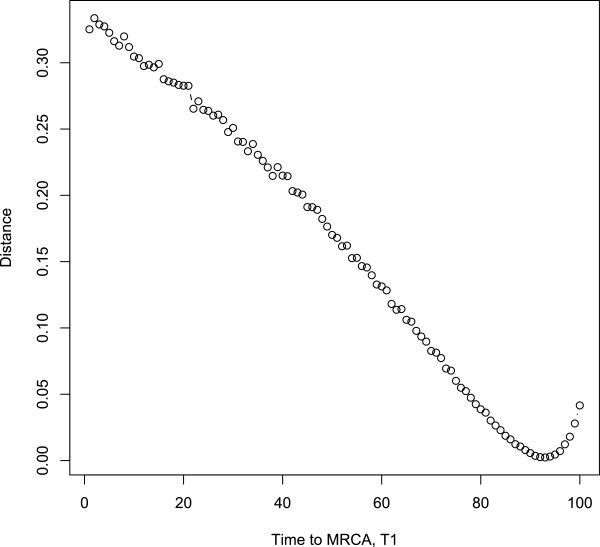
**Optimization of the tuning parameter*α*.***α* represents the time proportion from the earliest mutation event of the sample to the MRCA. *x*-axis represents *α* values and *y*-axis represents the square distance between the marginal distribution and the observed frequency of genotype data. Optimal *α* is 0.92 which minimizes the square distance represented in *y*-axis.

Figure 2 shows the marginal likelihood as a function of time to the MRCA from the earliest mutation event of the sample. The proportion of time from the earliest mutation event of the sample to the MRCA (*α*) was estimated as 0.92, based on the full dataset with 712 sites and 58 samples (for the details of the estimation method, see the Methods section below). This means that the proportion of time from the MRCA to the present day sequenced cells is around 8% of the total time. Note that the time proportion does not depend on any particular subset of sites, because it is optimized with the full 712 sites. The estimate does, however, depend on the number of cells selected for sequencing. There may be lineages of minor clones of cells not sequenced, whose MRCA occurred earlier.

Navin and Hicks [15] suggested several types of clonal evolutionary models in terms of diverse phylogenetic tree structures. Based on our estimation of the time to the MRCA of the samples, the time elapsed from the MRCA to the 58 sampled cells takes only 8% of the total time from the earliest mutation event to the sampling of cells. This indicates that most of the mutations in the 18 selected sites may be relatively late events in the history of the tumor. It also means that if represented in a genealogical tree, the branch between the root node and the MRCA takes up around 92% of the total height of the tree. This type of tree, having a long single branch from the root node, corresponds to the monogenomic tumor in Figure 1 of Navin and Hicks’ study.

## Conclusions and discussion

We have developed a new computational method to help elucidate aspects of the evolutionary history of a tumor based on single cell sequencing data. Cancer development is often characterized by the multistage progression of tumor sub-clones. Furthermore, the order of mutations among genes and pathways can play an important role in understanding tumor progression [16]. We have proposed a computational model to infer this mutation history, and our approach can be used to help understand those relationships. It is, however, challenging to infer the temporal ordering of mutational sites using current single cell sequencing technology. Effective reconstruction of the order of key mutational events is limited by the number of cells sequenced, and by the accuracy of the single cell sequences, which is an inherent limitation of current single cell sequencing data.

Single cell sequencing technologies generally require multiple steps, unique from bulk NGS technology, prior to DNA sequencing and conventional bioinformatic applications. These steps include cell isolation, cell lysis, and the amplification of the sampled cell’s DNA contents. Each step is susceptible to errors, such as contamination. Among them, the main limitation in current single cell sequencing is the process of amplification [17]. Since single cell sequencing relies on the tiny amount of genetic content of a single cell; unlike bulk multi-cell sequencing, the amplification of the DNA content of the sampled cell is critical. Various whole genome amplification techniques for single cell sequencing have been developed [18-20], some of which use PCR-free libraries. The dataset [2] used in our analysis is based on the multiple displacement amplification (MDA) technique [18]. Whole genome amplification often results in copying errors for individual bases, and in a failure to amplify larger regions. The false discovery rate resulting from copying errors of individual bases was quite low, but the allelic dropout rate resulting from the uneven distribution of amplification was very large in Hou et al.’s [2] MDA study.

Our likelihood function-based method incorporates the probabilities of both false discovery and allelic dropout error. That does not mean, however, that our estimates of pairwise ordering for mutation sites are correct. The probability that a pairwise ordering is correct depends on the number of cells sequenced. We conjecture that the number of cells one needs to sequence to achieve a desired ordering accuracy depends on the sequencing error rate. We plan to explore this issue in the future, but we expect that sequencing a large number of cells and reducing the allelic dropout rate are both important for correctly reconstructing the temporal order of mutations in a tumor.

### A Bayesian approach for inferring pairwise mutation orders

We constructed a mutation tree that shows the mutation order relations among the DNA sequence mutation sites of interest, based on the pairwise order relations. Additionally, we proposed a method for estimating the proportion of time from the earliest mutation event of the samples to the MRCA of the samples. This can provide useful information on inferring the clonal evolution of the tumor. We employed a Bayesian approach to determine the pairwise order relationship between any two mutation sites. The prior probability of the pairwise order of two mutation sites was computed by generating random genealogies and random mutations on the branches of the genealogies. The probability was then optimized to minimize its discrepancy from the observed pairwise frequency in an empirical Bayes manner. Sequencing errors were subsequently incorporated to compute the likelihood of pairwise orders so that the posterior probability was computed using Bayes theorem. The pairwise order relationships were determined based on the posterior probabilities. Pairwise orders obtained this way appeared robust, as tested by the leave-one-out replication of samples described earlier (see Figure 1). We also performed the site leave-one-out replication for the 18 sites, and found that the pairwise order relationships appeared robust (see Additional file 1 for details).

### Constructing the mutation tree

We constructed a mutation tree describing sequential mutation orders among sites based on pairwise order relationships. The minimal spanning tree algorithm [21] was used with negative log-posterior probabilities between two mutation sites for branch weights. Thus, the mutation tree we obtained can be regarded as a maximum likelihood tree among all possible trees, since the tree has the minimum total branch weights. We can infer the order relationship between any two distant sites ordered by branches in the tree. Under the assumption of one mutation per site, it is likely that mutation with higher mutation rate should occur earlier than mutation with lower mutation rate [22]. As shown in Figure 1, our mutation tree is sensible in that most branches are well aligned to the mutation rates for the 18 sites.

An alternate approach exists for constructing a mutation tree from a complex directed graph of pairwise mutation orders, given an ideal situation with no missing data and no sequencing errors. In this ideal situation, on could directly use the transitive property of the pairwise order relationships, without relying on the minimal spanning tree approach. For example, any three relations *x*→*y*,*y*→*z*, and *x*→*z* on three sites, *x*,*y*, and *z* can be reduced to *x*→*y* and *y*→*z* because the relation *x*→*z* is implied by the preceding two relations based on the transitive property. A tree constructed based on the transitive property can be interpreted more naturally than the likelihood-based minimal spanning tree. However, in a dataset with sequencing errors and missing entries, circular order relationships (for example, *x*→*y*,*y*→*z*, and *z*→*x*) often occur, and are not resolved using the transitive property. Thus, the likelihood-based minimal spanning tree approach is appropriate for our dataset (from Hou et al. [2]).

### Prior distribution to pairwise mutation orders

The coalescent tree model for computing prior probabilities of pairwise mutation orders assumes a constant population size. Since it is generally known that tumor size is not constant, but increases over time, we needed to evaluate whether our constant population size model fit our dataset adequately. Therefore, we performed additional simulations applying coalescent tree models with varying population growth rates. Population size is constant up to the MRCA of the samples in the model, and grows exponentially with a constant rate from the MRCA of the sample of cells (for the mathematical description, see Section 2.4 of Tavaré [23]). We found that optimized prior probabilities for various population size models are similar to those in the constant population size model (see Additional file 1). Thus, we concluded that the effects of population expansion for this dataset were small.

### Future directions

In summary, there are inherent challenges in using single cell sequencing data to elucidate the evolutionary relationships of a tumor. First, the error rates in single cell sequencing technology are generally high, and those error rates can impose considerable uncertainty on the base calling data, which may hinder proper analysis. We expect that these errors will be reduced by advances in single cell sequencing technology in the near future. Second, the limited number of samples in a dataset may not cover some lineages containing important mutations for tumor development. Consequently, it may result in a mutation tree in which important lineages are omitted. Therefore, a substantial number of cells are necessary to construct an optimal mutation tree for a tumor using single cell sequencing data. Determining the appropriate numbers of samples for analyzing single cell sequencing data is an important topic for future research.

In the future, we plan to investigate possible extensions of our tree model to different types of genomic data. For example, the approach can potentially be extended and applied to copy number variation data, as in Navin et al.’s work [3]. Alternate tree construction algorithms based on traditional phylogenetic methods or cell lineage analysis (for example, see Frumkin et al. [24]) for use with our tree model may also be useful.

## Methods

## Mutation ordering

To construct a tree describing temporal relationships of mutations, we begin by determining a partial order relationship for each pair of mutation sites. Directly constructing the joint order relations of all sites simultaneously increases combinatorial complexity exponentially and becomes computationally infeasible even for moderate number of mutation sites.

In order to determine the pairwise order relation, we consider the genealogy as a tree of the sample of sequenced cells. The tree traces how the sample cells evolved from the time when the first mutation of the samples occurred. The terminal nodes of the tree correspond to the sample cells in the dataset. The internal nodes in the tree correspond to the common ancestors of the lineages of the samples. Mutation events are superimposed on branches of the tree. Although the genealogical tree is different from the mutation tree we construct using the minimal spanning tree algorithm, the order relationships between two sites is best understood in terms of two mutation events in the genealogical tree.

In the genealogical tree of cell lineages, three order relations of mutation events of two sites *x*,*y* are possible; if mutation at *x* and mutation at *y* occur in the same lineage, then the two mutations have ancestral relationship. Otherwise, those two mutations have an independent relationship. So, we will denote the three partial order relations as *x*→*y*, *x*←*y*, *x*⇎*y* where for example, *x*→*y* represents mutation at *x* occurs earlier than the mutation at *y* in a lineage. The idea of clonal ordering [25] to determine the order of occurrences of neoplastic lesions is similar to the mutation ordering here.

### A simple example

We first illustrate how to determine the order of two mutation sites in a simple example. Consider a sample of 7 DNA sequences for two sites, *x* and *y* as in Table 2. Each site has only two variants, 0 as homozygous wildtype and 1 as heterozygous mutation type.

**Table 2:** **A simple genotype data with 7 samples for 2 sites,** ***x* and** ***y***

**Sample index**	**1**	**2**	**3**	**4**	**5**	**6**	**7**
Genotype at *x*	0	0	0	0	1	1	0
Genotype at *y*	0	0	1	1	1	1	1

0 indicates homozygous wildtype and 1 indicates heterozygous mutation.

If there are no sequencing errors in the data and mutation occurs once per site, then the temporal order of the mutations at the two sites *x* and *y* can be determined by the following reasoning. First, the genotype pair (1,1) in the data indicates that the mutations at *x* and *y* are ordered either as *x*→*y* or *x*←*y* because both mutations occurred in the lineages of samples 5 and 6. Second, the genotype pair (0,1) is not compatible with the relation *x*→*y* because the relation means that a mutation at *y* occurs in the same lineage and after a mutation at *x* occurs. Consequently, we determine that the mutation at *y* is ancestral to the mutation at *x*. Simply, one could determine the order relation by examining whether the set of samples with mutation at one site are included in the set of samples with mutation at the other site as for the gene tree algorithm of [5] and [26].

**Figure 3:**
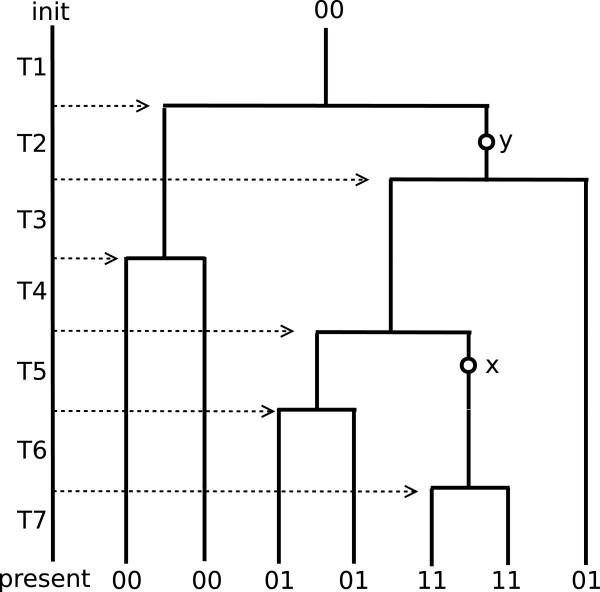
**The tree represents one possible genealogy generating the data of Table**1**.** The seven samples with integer genotype pairs at *x* and *y* are attached to the leaves of the genealogical tree. Initial genotype pair at the root is assumed as 00, wildtype at *x* and wildtype at *y*. The left line represents the time from the earliest mutation event of the sample (as “init”) to the current sample (as “present”). The seven time intervals are illustrated in the context of coalescent tree of the seven samples. From the leaves to the root, the six coalescent events determine the time intervals, *T*1,…,*T*7. Two mutations at *x* and *y* occurred in time interval *T*5 and *T*2, respectively which are marked as circles with “x” and “y”.

Figure 3 shows one possible genealogy which generates the genotype data in Table 2. The tree describes how the genotype pairs at the two sites *x* and *y* of the sample of 7 cells were generated from the initial wildtype pair (0,0) at *x* and *y*. In the figure, mutation at *y* is ancestral to mutation at *x* in the lineages of two sample cells.

The example above illustrates how to determine the pairwise order relationship of two mutation sites. However, in a real dataset such a simple deterministic rule is inadequate as there are many incompatible cases, for example two mutation sites having the genotypes (0,1),(1,0),(1,1). Homozygous mutations (mutations occurring in both alleles) are common due to high sequencing error rates. In order to deal with such problems, we need a probabilistic approach to incorporate those features of single cell sequencing data to our model.

### Proposed method

We propose a Bayesian approach to determine the pairwise mutation order of two base pair sites. Directly optimizing the order relationships among all sites is computationally infeasible but the mutation tree can be estimated based on the pairwise order relationships.

Let *L*(*x*∼*y*) denote the likelihood of the data *D* when the mutations are in relation *x*∼*y*. *x*∼*y* can be either *x*→*y*, *x*←*y* or *x*⇎*y*. The data *D* consist of the bivariate genotypes at sites *x* and *y* for all samples, i.e. *D*={(*i*_*k*_,*j*_*k*_),*k*=1,…,*n*} where *i*_*k*_ denotes the observed genotype of the *k*th sample at site *x*. *i*_*k*_=0 denotes that sample *k* is homozygous wildtype, *i*_*k*_=1 denotes heterozygous mutation, and *i*_*k*_=2 denotes homozygous mutation at *x*. The likelihood can be written as

(1) $$\begin{array}{l} L(x∼y)=\overset{n}{\underset{k=1}{\prod }}\mathrm{Pr}((i_{k},j_{k})\;|\;x∼y) \end{array}$$

The likelihood function reflects both the restrictions imposed by the order relationship and sequencing errors. These can be decomposed as

(2) $$\begin{array}{l} \;\mathrm{Pr}((i,j)\;|\;x∼y)=\underset{i^{\prime },\;j^{\prime }=\;0,1}{\sum }\mathrm{Pr}((i,j)\;|\;(i^{\prime },j^{\prime }))\mathrm{Pr}((i^{\prime },j^{\prime })\;|\;x∼y) \end{array}$$

where (*i*^′^,*j*^′^) are the true genotypes at *x* and *y*. The left hand side of (2) is the probability of getting sequencing data (*i*,*j*) if the true order relationship is *x*∼*y*. The true genotypes (*i*^′^,*j*^′^) can be (0,0),(0,1),(1,0) or (1,1) where, for example, (0,0) means that both sites are homozygous wildtype. The second factor under the summation is the probability that the true genotypes are (*i*^′^,*j*^′^) given that the order relationship is *x*∼*y*. The first factor under the summation is the probability of getting the sequence data (*i*,*j*) given that the true genotypes are (*i*^′^,*j*^′^). This obviously depends on the accuracy of the sequencing assay. Suppose the true genotypes are (1,1), meaning both sites have heterozygous mutations. Then the sequencing data could be (0,0) if allelic dropout sequencing error occurs at both sites. If the genotypes are (0,1), then the sequencing data could be (0,0) if an allelic dropout occurs at the second site and no false discovery occurs at the first site.

The first factor under the summation can be computed based on the probability of allelic dropout and false discoveries. The second factor represents four unknown probabilities (which sum to 1) for each order relationship. To avoid overfitting the data we use a Bayesian approach.

Using Bayes theorem,

(3) $$\begin{array}{l} \mathrm{Pr}(x∼y\;|\;D)\propto L(x∼y)\mathrm{Pr}(x∼y) \end{array}$$

where *P*(*x*∼*y*) denotes the prior probability of *x*∼*y*. Expression (3) provides a quantification of the evidence for each of the possible order relationships.

#### ***Prior model of mutation order***

For any pair of mutation site (*x*,*y*) we need to compute the prior probabilities that *x*→*y*,*x*←*y* and *x*⇎*y* in the following way. 

1. Generate a random binary genealogical tree with *n* terminal nodes (for example, see Figure 3).

2. Generate time intervals *T*_1_,…,*T*_*n*_ where *T*_*k*_ denotes the time during which *k* distinct lineages exist in the tree. *T*_*k*_ represents the time till the (*n*-*k*+1)th coalescent event from the (*n*-*k*)th coalescent event. *T*_*k*_ is exponentially distributed with the rate parameter, k2[27]. Given *T*_2_,…,*T*_*n*_ values, *T*_1_ is computed from the relation, $T_{1}/\overset{n}{\underset{i=1}{\sum }}\text{Ti}=\alpha$ (See below for specification of *α*).

3. Generate *B*_mut_ independent pairs of (*x*,*y*) mutations in the binary genealogical tree specified in steps 1 and 2. Each mutation occurs randomly at a time uniformly distributed between 0 and *T*_1_+⋯+*T*_*n*_. The branch on which each mutation occurs is selected randomly from the branches that exist at that time. Thus the probability of a mutation occurring in *T*_*k*_ and one of the *n* lineages is $T_{k}/\overset{n}{\underset{i=1}{\sum }}i\cdot T_{i}$.

4. Repeat Step 1 to 3 for *B*_tree_ times.

In our simulations, the number of trees generated *B*_tree_ was 1000 and the number of mutation pairs per tree *B*_mut_ was 10000 for Step 2.

The prior probability of mutation orders are then computed by counting the corresponding cases as

(4) $$\;\begin{array}{c} \mathrm{Pr}(x\to y)=\mathrm{Pr}(x\leftarrow y)\\ \;=\frac{1}{2}\cdot \frac{\text{No. of mutation pairs on same lineage}}{B_{\text{tree}}\cdot B_{\text{mut}}},\\ \;\mathrm{Pr}(x⇎y)=1-2\mathrm{Pr}(x\to y). \end{array}$$

For computing the likelihood in (1), the probability of true genotype (*i*^′^,*j*^′^) given *x*∼*y* is

(5) $$\begin{array}{l} \mathrm{Pr}((i^{\prime },j^{\prime })\;|\;x∼y)=\frac{\text{No. of cases of}(i^{\prime },j^{\prime })\text{when}x∼y}{\text{No. of cases with}x∼y} \end{array}$$

where *i*^′^,*j*^′^ are either 0 or 1.

The parameter *α* in Step 2 specifies the proportion of the time from the earliest mutation event of the tumor to the MRCA of the samples. In the simulation above, multiple *α* values in the unit interval are tried and the value which maximizes the likelihood is used. The optimization procedure is described in the section below (Tuning prior model and time estimation to the MRCA). The time intervals *T*_2_,…,*T*_*n*_ in Step 2 are assumed exponentially distributed based on the genealogy of the neutral Wright-Fisher model [23].

In the prior model, only homozygous wildtype (encoded as 0) and heterozygous mutation (encoded as 1) genotypes are used to denote the true genotypes *i*^′^ and *j*^′^. Homozygous mutation at a base pair site is not used in the prior model because it is extremely unlikely [2]. However, homozygous mutations are observed in the real dataset because of sequencing errors.

#### ***Sequencing errors and likelihood computation***

We consider two kinds of sequencing errors which transform the true genotypes to the observed genotypes. One is the error of calling heterozygous, a homozygous site and the other is the error of calling homozygous, a heterozygous site. The former is called false discovery and the latter is called allelic dropout in [2]. We denote *FD* and *AD* as the error rates for false discovery and allelic dropout, respectively, which mean

$$\begin{array}{l} \text{FD}=\mathrm{Pr}(i=1\;|\;i^{\prime }=0),\;\text{AD}=\mathrm{Pr}(i=0\text{or}2\;|\;i^{\prime }=1) \end{array}$$

for the observed genotype *i* and its corresponding true genotype *i*^′^. We adopted the values 6.04×10^-5^ and 0.4309 for *FD* and *AD* from [2].

The *FD* and *AD* are used to compute the likelihood of observed genotypes in (2) and (1). The probability of the observed genotype (*i*,*j*) at *x*,*y* given true genotype (*i*^′^,*j*^′^) in (2) is decomposed as Pr((*i*,*j*) | (*i*^′^,*j*^′^))= Pr(*i* | *i*^′^) Pr(*j* | *j*^′^) assuming the errors occur independent of the mutation sites. For each pair of *i* and *i*^′^, we use

$$\begin{array}{lll} \mathrm{Pr}(i=1\;|\;i^{\prime }=0) & =\text{FD},\; & \;\\ \mathrm{Pr}(i=2\;|\;i^{\prime }=0) & =c,\; & \;\\ \mathrm{Pr}(i=0\;|\;i^{\prime }=0) & =1-\text{FD}-c,\; & \;\\ \mathrm{Pr}(i=0\;|\;i^{\prime }=1) & =\mathrm{Pr}(i=2\;|\;i^{\prime }=1)=\frac{\text{AD}}{2},\; & \;\\ \mathrm{Pr}(i=1\;|\;i^{\prime }=1) & =1-\text{AD}\; & \; \end{array}$$

to compute the factor in the decomposition. Pr(*i* = 2 | *i*^′^ = 0) is not determined based on *FD* and *AD* but we assume it is negligible because it is likely to be much smaller than *FD*=6.04×10^-5^.

#### ***Tuning prior model and time estimation to the MRCA***

We estimate *α*, the proportion of time from the earliest mutation to the MRCA of the sampled cells:

(6) $$\;\alpha =\frac{\text{time from the earliest mutation to the MRCA}}{\text{total time from the earliest mutation to the sample}}.$$

*α* is the only parameter which is optimized in the prior model. We estimate *α* by an empirical Bayes method which is based on the comparison between the marginal genotype distribution and the relative frequency of observed genotypes of the full dataset. The marginal probability of the observed genotype (*i*,*j*) is

$$\begin{array}{l} p_{\text{ij}}=\underset{x∼y}{\sum }\mathrm{Pr}((i,j)\;|\;x∼y)\mathrm{Pr}(x∼y). \end{array}$$

The right hand side of the above equation performs the summation over the three order relations. If we let *f*_*ij*_ be the relative frequency of the observed genotype (*i*,*j*), then the parameter *α* is optimized to minimize the distance between the two quantities:

(7) $$\hat{\alpha }=\arg\underset{\alpha }{\min}\underset{i,\;j=0,1,2}{\sum }{\left(p_{\text{ij}}-f_{\text{ij}}\right)}^{2}.$$

## Mutation tree

The mutation order relation between two sites is determined by selecting the maximum of the three posterior probabilities calculated using (3) for the two sites. Based on the mutation sites and determined order relations, we can construct a directed graph. In the directed graph, each node corresponds to a mutation site and direction of each branch corresponds to a mutation order. In general the directed graph is not a tree but forms a complex network of order relationships. If the relation between two sites with maximum posterior probability is ⇎, then there is no branch between the sites.

Because it is difficult to interpret order relationships among sites directly from this directed graph, we extract a tree structure from the directed graph to clarify key relationships among sites. We construct a minimal spanning mutation tree based on the directed graph. To apply the minimal spanning tree algorithm [21], we assign weights on branches in the directed graph. The weight of branch *w* in the graph is encoded as - log Pr(*x*∼*y* | *D*) where *x*∼*y* is the order relation between *x* and *y* in the graph and *D* denotes the genotype data. An optimum tree $\hat{T}$ is then sought with minimum total weight,

$$\;\hat{T}=\arg\underset{T}{\min}\underset{x∼y\in T}{\sum }w_{x∼y}=\arg\underset{T}{\max}\underset{x∼y\in T}{\prod }\mathrm{Pr}(x∼y\;|\;D).$$

This tree has maximum posterior probability among all possible trees.

**Figure 4:**
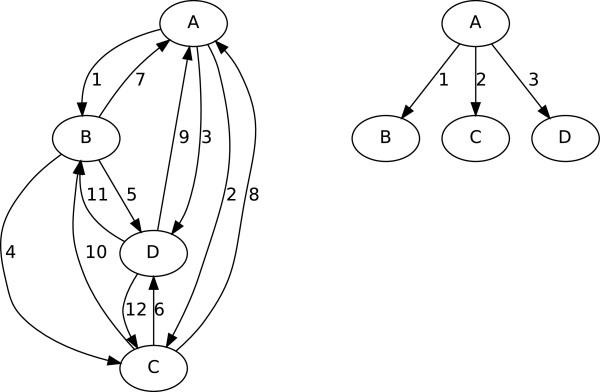
**An example of minimal spanning tree of a directed graph.** A directed graph consisting of four nodes, ’A’, ’B’, ’C’ and ’D’ is shown in the left hand side. Nodes in the directed graph are completely connected to each other with integer weights ranging from 1 to 12. In the right hand side, the corresponding minimal spanning tree of the directed graph is shown. The tree has the minimum total weight among all possible trees contained in the directed graph.

Figure 4 illustrates a simple example of a directed graph and the corresponding minimal spanning tree. The minimal spanning tree of the directed graph is a tree with all the nodes in the directed graph. Branches in the minimal spanning tree are selected to minimize the total sum of the weights in the branches. In the figure, the total weight of the tree is 6 which is the minimum total weights of branches among all possible trees from the directed graph.

## Availability

Source codes are available at https://sites.google.com/site/kyungin2013/home/muttree-codes.tar.gz.

## Competing interests

The authors declare that they have no competing interests.

## Authors’ contributions

KIK and RS conceived the study. KIK developed and implemented the algorithm. KIK and RS wrote the manuscript. Both authors read and approved the final manuscript.

## Supplementary Material

Additional file 1 **Supplementary Information.** Additional file 1 consists of two figures and two tables. The two figures are stability of mutation tree of the 18 sites and optimized time to the MRCA under varying population growth rate. The two tables are posterior probabilities of pairwise order relations for the 18 sites and prior probabilities under varying population growth rate. Click here for file

## References

[B1] Tsao JL Yatabe Y Salovaara R Järvinen HJ Mecklin JP Aaltonen LA Tavaré S Shibata D **Genetic reconstruction of individual colorectal tumor histories** Proc Nat Acad Sci 2000 97 3 1236 1241 [http://www.pnas.org/content/97/3/1236.abstract] 10.1073/pnas.97.3.1236 10655514 PMC15581

[B2] Hou Y Song L Zhu P Zhang B Tao Y Xu X Li F Wu K Liang J Shao D Wu H Ye X Ye C Wu R Jian M Chen Y Xie W Zhang R Chen L Liu X Yao X Zheng H Yu C Li Q Gong Z Mao M Yang X Yang L Li J Wang W **Single-cell exome sequencing and monoclonal evolution of a, JAK2-negative myeloproliferative neoplasm** Cell 2012 148 5 873 885 10.1016/j.cell.2012.02.028 22385957

[B3] Navin N Kendall J Troge J Andrews P Rodgers L McIndoo J Cook K Stepansky A Levy D Esposito D Muthuswamy L Krasnitz A McCombie WR Hicks J Wigler M **Tumour evolution inferred by single-cell sequencing** Nature 2011 472 7341 90 94 10.1038/nature09807 21399628 PMC4504184

[B4] Li Y Xu X Song L Hou Y Li Z Tsang S Li F Im K Wu K Wu H Ye X Li G Wang L Zhang B Liang J Xie W Wu R Jiang H Liu X Yu C Zheng H Jian M Nie L Wan L Shi M Sun X Tang A Guo G Gui Y Cai Z **Single-cell sequencing analysis characterizes common and cell-lineage-specific mutations in a muscle-invasive bladder cancer** GigaScience 2012 1 12 [http://www.gigasciencejournal.com/content/1/1/12] 10.1186/2047-217X-1-12 23587365 PMC3626503

[B5] Gusfield D **Efficient algorithms for inferring evolutionary trees** Networks 1991 21 19 28 [http://dx.doi.org/10.1002/net.3230210104] 10.1002/net.3230210104

[B6] Griffiths RC Tavaré S **Ancestral inference in population genetics** Stat Sci 1994 9 3 307 319 10.1214/ss/1177010378

[B7] Griffiths RC Tavaré S **The ages of mutations in gene trees** Ann Appl Probab 1999 9 3 567 590 [http://dx.doi.org/10.1214/aoap/1029962804]

[B8] Desper R Jiang F Kallioniemi OP Moch H Papadimitriou CH Schaffer AA **Inferring tree models for oncogenesis from comparative genome hybridization data** J Comput Biol 1999 6 37 51 10.1089/cmb.1999.6.37 10223663

[B9] Kumar P Henikoff S Ng PC **Predicting the effects of coding non-synonymous variants on protein function using the SIFT algorithm** Nat Protoc 2009 4 7 1073 1081 19561590 10.1038/nprot.2009.86

[B10] Futreal PA Coin L Marshall M Down T Hubbard T Wooster R Rahman N Stratton MR **A census of human cancer genes** Nat Rev Cancer 2004 4 3 177 183 10.1038/nrc1299 14993899 PMC2665285

[B11] R Core Team R: A Language and Environment for Statistical Computing 2012 Vienna: R Foundation for Statistical Computing [http://www.R-project.org/]. [ISBN 3-900051-07-0]

[B12] Carey V Gentleman R Long L RBGL: An Interface to the BOOST Graph Library. 2005 [http://www.bioconductor.org]. [R package version 1.32.1]

[B13] Gansner ER North SC **An open graph visualization system and its applications to software engineering** Softw - Pract Exp 2000 30 11 1203 1233 10.1002/1097-024X(200009)30:11<1203::AID-SPE338>3.0.CO;2-N

[B14] Sablina AA Budanov AV Ilyinskaya GV Agapova LS Kravchenko JE Chumakov PM **The antioxidant function of the p53 tumor suppressor** Nat Med 2005 11 12 1306 1313 10.1038/nm1320 16286925 PMC2637821

[B15] Navin NE Hicks J **Tracing the tumor lineage** Mol Oncol 2010 4 3 267 283 10.1016/j.molonc.2010.04.010 20537601 PMC2904844

[B16] Weinberg RA The Biology of Cancer 2006 New York: Garland Science

[B17] Blainey PC **The future is now: single-cell genomics of bacteria and archaea** FEMS Microbiol Rev 2013 37 3 407 427 10.1111/1574-6976.12015 23298390 PMC3878092

[B18] Dean FB Nelson JR Giesler TL Lasken RS **Rapid amplification of plasmid and phage DNA using Phi 29 DNA polymerase and multiply-primed rolling circle amplification** Genome Res 2001 11 6 1095 1099 10.1101/gr.180501 11381035 PMC311129

[B19] Telenius H Carter NP Bebb CE Nordenskjold M Ponder BA Tunnacliffe A **Degenerate oligonucleotide-primed PCR: general amplification of target DNA by a single degenerate primer** Genomics 1992 13 3 718 725 10.1016/0888-7543(92)90147-K 1639399

[B20] Zong C Lu S Chapman AR Xie XS **Genome-wide detection of single-nucleotide and copy-number variations of a single human cell** Science 2012 338 6114 1622 1626 10.1126/science.1229164 23258894 PMC3600412

[B21] Edmonds J **Optimum branchings** J Res Nat Bur Stand Sect B 1967 71B 233 240 10.6028/jres.071B.032

[B22] Yeang CH McCormick F Levine A **Combinatorial patterns of somatic gene mutations in cancer** FASEB J 2008 22 8 2605 2622 10.1096/fj.08-108985 18434431

[B23] Tavaré S **Ancestral inference in population genetics** Lectures on Probability Theory and Statistics, Volume 1837 of Lecture Notes in Math 2004 Berlin: Springer 1 188

[B24] Frumkin D Wasserstrom A Itzkovitz S Stern T Harmelin A Eilam R Rechavi G Shapiro E **Cell lineage analysis of a mouse tumor** Cancer Res 2008 68 14 5924 5931 10.1158/0008-5472.CAN-07-6216 18632647

[B25] Merlo LMF Pepper JW Reid BJ Maley CC **Cancer as an evolutionary and ecological process** Nat Rev: Cancer 2006 6 924 935 10.1038/nrc2013 17109012

[B26] Griffiths RC Veuille M, Slatkin M **Ancestral inference from gene trees** Modern Developments in Theoretical Population Genetics: the Legacy of Gustave Malécot, Oxford Biology Readers 2002 New York: Oxford University Press 94 117

[B27] Hudson RR **Gene genealogies and the coalescent process** Oxford Surveys in Evolutionary Biology 1991 New York: Oxford University Press 1 44

