# Supplementary material for: Using single cell sequencing data to model the evolutionary history of a tumor
Source: BMC Bioinformatics. 2014 Jan 24;15:27. doi: 10.1186/1471-2105-15-27 (PMC3903814; doi:10.1186/1471-2105-15-27)
Supplement: Additional file 1 — Supplementary Information. Additional file 1 consists of two figures and two tables. The two figures are stability of mutation tree of the 18 sites and optimized time to the MRCA under varying population growth rate. The two tables are posterior probabilities of pairwise order relations for the 18 sites and prior probabilities under varying population growth rate. [file 1471-2105-15-27-S1.pdf]

Supplementary Information of "Using Single Cell Sequencing Data to Model the Evolutionary History of a Tumor"

Kyung In Kim and Richard Simon

November 11, 2013

1 Stability of mutation tree of the 18 sites

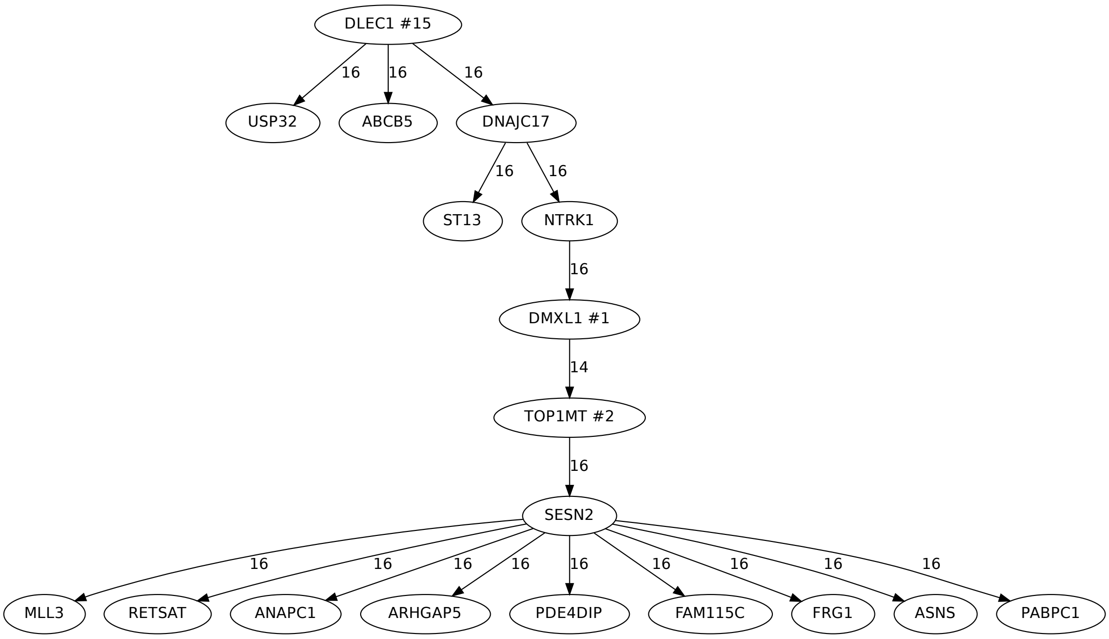

Figure S1: Summary of the site leave-one-out replication for the 18 sites. Each tree was based on 17 sites, one site omitted from the 18 sites sequentially. The frequencies of root site and branches of the site leave-one-out tree in the original tree are presented. For example, DLEC1 was found as root in 15 trees and DMXL1 was found as root in 1 trees and TOP1MT was found as root in 2 trees. Branches in the original tree were also found frequently in the 18 trees and the minimum frequency of branch in the original tree was 14.

2 Posterior probabilities of pairwise order relations for the 18 sites

Table S1: Posterior probabilities of pairwise order relations for the 18 sites.

site x	site y	$x \to y$	$x \leftarrow y$	$x \not\leftrightarrow y$
PDE4DIP	NTRK1	0.018892	0.981108	0
PDE4DIP	SESN2	9.9e-05	0.999901	0
PDE4DIP	ARHGAP5	0.499022	0.499038	0.00194
PDE4DIP	DNAJC17	0.000371	0.999629	0
PDE4DIP	USP32	0.981107	0.018893	0
PDE4DIP	ANAPC1	0.932969	0.067031	0
PDE4DIP	RETSAT	0.980556	0.018883	0.000561
PDE4DIP	ST13	0.005135	0.994865	0
PDE4DIP	DLEC1	0.018892	0.981108	0
PDE4DIP	FRG1	0.997664	0.00138	0.000956
PDE4DIP	DMXL1	0.067026	0.932974	0
PDE4DIP	FAM115C	0.932965	0.067029	7e-06
PDE4DIP	MLL3	0.578722	0.002987	0.418291
PDE4DIP	ABCB5	0.211371	0.788629	0
PDE4DIP	ASNS	0.784308	0.210225	0.005466
PDE4DIP	PABPC1	0.149453	0.000771	0.849776
PDE4DIP	TOP1MT	0.000371	0.999629	0
NTRK1	SESN2	0.001382	0.998618	0
NTRK1	ARHGAP5	0.981108	0.018892	0
NTRK1	DNAJC17	0.000371	0.999629	0
NTRK1	USP32	0.999993	7e-06	0
NTRK1	ANAPC1	0.981108	0.018892	0
NTRK1	RETSAT	0.999629	0.000371	0
NTRK1	ST13	0.788644	0.211356	0
NTRK1	DLEC1	0.067026	0.932974	0
NTRK1	FRG1	1	0	0
NTRK1	DMXL1	0.932967	0.067033	0
NTRK1	FAM115C	0.932965	0.067029	7e-06
NTRK1	MLL3	0.999901	9.9e-05	0
NTRK1	ABCB5	0.981107	0.018893	0
NTRK1	ASNS	0.999973	2.7e-05	0
NTRK1	PABPC1	0.999989	7e-06	3e-06
NTRK1	TOP1MT	0.005135	0.994865	0
SESN2	ARHGAP5	1	0	0
SESN2	DNAJC17	0.067031	0.932969	0
SESN2	USP32	0.999629	0.000371	0
SESN2	ANAPC1	0.999998	2e-06	0
SESN2	RETSAT	1	0	0
SESN2	ST13	0.994866	0.005134	0
SESN2	DLEC1	0.211371	0.788629	0
SESN2	FRG1	1	0	0

Table S1

site x	site y	$x \to y$	$x \leftarrow y$	$x \not\leftrightarrow y$
SESN2	DMXL1	0.499988	0.500012	0
SESN2	FAM115C	0.932974	0.067026	0
SESN2	MLL3	1	0	0
SESN2	ABCB5	0.788614	0.211386	0
SESN2	ASNS	1	0	0
SESN2	PABPC1	1	0	0
SESN2	TOP1MT	0.067027	0.932973	0
ARHGAP5	DNAJC17	7e-06	0.999993	0
ARHGAP5	USP32	0.99445	0.005133	0.000417
ARHGAP5	ANAPC1	0.211385	0.788615	0
ARHGAP5	RETSAT	0.981107	0.018893	0
ARHGAP5	ST13	0.000371	0.999629	0
ARHGAP5	DLEC1	9.9e-05	0.999901	0
ARHGAP5	FRG1	0.000262	0	0.999737
ARHGAP5	DMXL1	0.001382	0.998618	0
ARHGAP5	FAM115C	0.003607	0.050203	0.94619
ARHGAP5	MLL3	0.994736	0.005134	0.00013
ARHGAP5	ABCB5	0.067027	0.932973	0
ARHGAP5	ASNS	0.932973	0.067027	0
ARHGAP5	PABPC1	0.999838	9.9e-05	6.3e-05
ARHGAP5	TOP1MT	0.000371	0.999629	0
DNAJC17	USP32	0.999901	9.9e-05	0
DNAJC17	ANAPC1	0.999993	7e-06	0
DNAJC17	RETSAT	0.999999	1e-06	0
DNAJC17	ST13	0.999629	0.000371	0
DNAJC17	DLEC1	0.018891	0.981109	0
DNAJC17	FRG1	1	0	0
DNAJC17	DMXL1	0.78861	0.21139	0
DNAJC17	FAM115C	0.932973	0.067027	0
DNAJC17	MLL3	0.999999	1e-06	0
DNAJC17	ABCB5	0.788615	0.211385	0
DNAJC17	ASNS	1	0	0
DNAJC17	PABPC1	1	0	0
DNAJC17	TOP1MT	0.211375	0.788625	0
USP32	ANAPC1	0.499992	0.500008	0
USP32	RETSAT	0.497166	0.497166	0.005669
USP32	ST13	9.9e-05	0.999901	0
USP32	DLEC1	7e-06	0.999993	0
USP32	FRG1	0.981106	0.018893	0
USP32	DMXL1	0.005135	0.994865	0
USP32	FAM115C	0.788615	0.211385	0
USP32	MLL3	0.73977	0.198287	0.061943
USP32	ABCB5	0.001382	0.998618	0
USP32	ASNS	0.932903	0.067024	7.3e-05
USP32	PABPC1	1e-05	0	0.99999
USP32	TOP1MT	0.001381	0.998619	0
ANAPC1	RETSAT	0.999629	0.000371	0

Table S1

site x	site y	$x \to y$	$x \leftarrow y$	$x \not\leftrightarrow y$
ANAPC1	ST13	9.9e-05	0.999901	0
ANAPC1	DLEC1	0.001381	0.998619	0
ANAPC1	FRG1	0.549926	0.039508	0.410565
ANAPC1	DMXL1	0.067026	0.932974	0
ANAPC1	FAM115C	0.118776	0.443143	0.438082
ANAPC1	MLL3	0.980448	0.01888	0.000672
ANAPC1	ABCB5	0.000371	0.999629	0
ANAPC1	ASNS	0.932939	0.067027	3.4e-05
ANAPC1	PABPC1	0.111167	1.1e-05	0.888822
ANAPC1	TOP1MT	9.9e-05	0.999901	0
RETSAT	ST13	2e-06	0.999998	0
RETSAT	DLEC1	2.7e-05	0.999973	0
RETSAT	FRG1	0.000111	8e-06	0.999881
RETSAT	DMXL1	2.7e-05	0.999973	0
RETSAT	FAM115C	0.208083	0.776318	0.015598
RETSAT	MLL3	0.065852	0.916589	0.017559
RETSAT	ABCB5	0.005135	0.994865	0
RETSAT	ASNS	0.003709	0.718481	0.27781
RETSAT	PABPC1	3e-06	0	0.999997
RETSAT	TOP1MT	9.9e-05	0.999901	0
ST13	DLEC1	0.211361	0.788639	0
ST13	FRG1	1	0	0
ST13	DMXL1	0.788595	0.211405	0
ST13	FAM115C	0.499993	0.500007	0
ST13	MLL3	0.999998	2e-06	0
ST13	ABCB5	0.49998	0.50002	0
ST13	ASNS	0.999901	9.9e-05	0
ST13	PABPC1	0.999994	0	6e-06
ST13	TOP1MT	0.01889	0.98111	0
DLEC1	FRG1	1	0	0
DLEC1	DMXL1	0.211381	0.788619	0
DLEC1	FAM115C	0.929712	0.066791	0.003497
DLEC1	MLL3	0.999973	2.7e-05	0
DLEC1	ABCB5	0.994865	0.005135	0
DLEC1	ASNS	0.999998	2e-06	0
DLEC1	PABPC1	1	0	0
DLEC1	TOP1MT	0.500008	0.499992	0
FRG1	DMXL1	7e-06	0.999989	4e-06
FRG1	FAM115C	0.210357	0.784802	0.004841
FRG1	MLL3	0	0	1
FRG1	ABCB5	0	1	0
FRG1	ASNS	0	0	1
FRG1	PABPC1	0	0	1
FRG1	TOP1MT	2e-06	0.999998	0
DMXL1	FAM115C	0.211385	0.788615	0
DMXL1	MLL3	0.999901	9.9e-05	0
DMXL1	ABCB5	0.788626	0.211374	0

Table S1

site x	site y	$x \to y$	$x \leftarrow y$	$x \not\leftrightarrow y$
DMXL1	ASNS	0.999629	0.000371	0
DMXL1	PABPC1	0.999865	0	0.000135
DMXL1	TOP1MT	0.788615	0.211385	0
FAM115C	MLL3	0.00844	0.000606	0.990954
FAM115C	ABCB5	0.067029	0.932971	0
FAM115C	ASNS	0.500007	0.499993	0
FAM115C	PABPC1	0.156302	0.00301	0.840688
FAM115C	TOP1MT	0.211381	0.788619	0
MLL3	ABCB5	2e-06	0.999998	0
MLL3	ASNS	0.018893	0.981104	3e-06
MLL3	PABPC1	0.000128	1e-06	0.999872
MLL3	TOP1MT	2.7e-05	0.999973	0
ABCB5	ASNS	0.999993	7e-06	0
ABCB5	PABPC1	0.999999	1e-06	0
ABCB5	TOP1MT	0.067027	0.932973	0
ASNS	PABPC1	0	0	1
ASNS	TOP1MT	0.000371	0.999629	0
PABPC1	TOP1MT	1e-06	0.999999	0

3 Optimized time to the MRCA under varying population growth rate

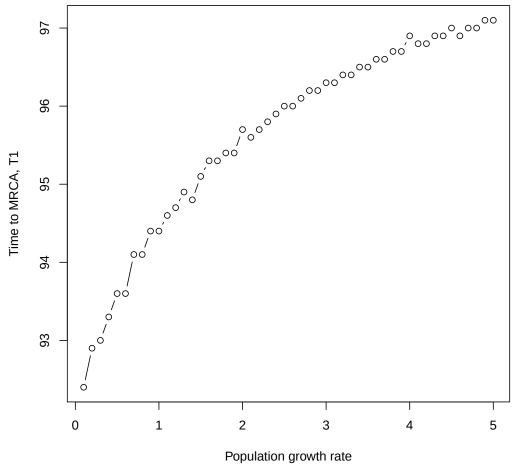

Figure S2: Optimization of the tuning parameter α under varying population growth rate. x-axis represents varying popoulation growth rates and y-axis represents the optimized proportion of time to the MRCA over the total time, α. Population size is assumed to increase with a constant rate from the MRCA of the sample. Under the constant population size (population growth rate = 0), the optimized α is 0.92. The optimized α appears to increase as the fixed population growth rate increases.

4 Prior probabilities under varying popoulation growth rate

Table S2: Prior probabilities, $\Pr(x \rightarrow y)$ and $\Pr(x \not\leftrightarrow)$ for given population growth rate. $\Pr(x \leftarrow y)$ is same as $\Pr(x \rightarrow y)$.

population growth rate	$\Pr(x \rightarrow y)$	$\Pr(x \not\leftrightarrow y)$
0.1	0.46452	0.07095
0.2	0.46495	0.07011
0.3	0.46481	0.07038
0.4	0.46471	0.07058
0.5	0.46515	0.06970
0.6	0.46447	0.07107
0.7	0.46544	0.06912
0.8	0.46520	0.06960
0.9	0.46496	0.07009
1.0	0.46452	0.07097
1.1	0.46458	0.07084
1.2	0.46528	0.06943
1.3	0.46465	0.07069
1.4	0.46406	0.07188
1.5	0.46546	0.06908
1.6	0.46492	0.07016
1.7	0.46512	0.06975
1.8	0.46448	0.07104
1.9	0.46442	0.07116
2.0	0.46510	0.06980
2.1	0.46457	0.07087
2.2	0.46505	0.06991
2.3	0.46429	0.07141
2.4	0.46492	0.07017
2.5	0.46591	0.06817
2.6	0.46537	0.06926
2.7	0.46542	0.06917
2.8	0.46561	0.06878
2.9	0.46470	0.07061
3.0	0.46398	0.07204
3.1	0.46409	0.07182
3.2	0.46513	0.06974
3.3	0.46491	0.07019
3.4	0.46495	0.07009
3.5	0.46464	0.07072
3.6	0.46465	0.07070
3.7	0.46457	0.07087
3.8	0.46501	0.06999
3.9	0.46530	0.06941
4.0	0.46669	0.06661
4.1	0.46545	0.06911
4.2	0.46415	0.07170
4.3	0.46555	0.06890
4.4	0.46520	0.06961
4.5	0.46513	0.06974
4.6	0.46415	0.07170
4.7	0.46559	0.06882
4.8	0.46422	0.07156
4.9	0.46580	0.06839
5.0	0.46462	0.07076

Table S3: Prior probabilities, $\Pr((0,0)\,|\,x \rightarrow y), \Pr((1,0)\,|\,x \rightarrow y)$ and $\Pr((1,1)\,|\,x \rightarrow)$ for given population growth rate. $\Pr((0,1)\,|\,x \rightarrow y) = 0$. For the relation $x \leftarrow y$, $\Pr((0,0)\,|\,x \leftarrow y) = \Pr((0,0)\,|\,x \rightarrow y), \Pr((0,1)\,|\,x \leftarrow y) = \Pr((1,0)\,|\,x \rightarrow y), \Pr((1,0)\,|\,x \leftarrow y) = \Pr((0,1)\,|\,x \rightarrow y) = 0$, and $\Pr((1,1)\,|\,x \leftarrow y) = \Pr((1,1)\,|\,x \rightarrow y)$.

population growth rate	$\Pr((0,0)\,|\,x \rightarrow y)$	$\Pr((1,0)\,|\,x \rightarrow y)$	$\Pr((1,1)\,|\,x \rightarrow y)$
0.1	0.01710	0.36741	0.61549
0.2	0.01660	0.36723	0.61616
0.3	0.01709	0.36717	0.61575
0.4	0.01674	0.36633	0.61693
0.5	0.01676	0.36588	0.61735
0.6	0.01729	0.36818	0.61453
0.7	0.01644	0.36403	0.61954
0.8	0.01694	0.36483	0.61822
0.9	0.01647	0.36558	0.61795
1.0	0.01707	0.36774	0.61518
1.1	0.01682	0.36739	0.61579
1.2	0.01669	0.36463	0.61869
1.3	0.01677	0.36809	0.61514
1.4	0.01723	0.36880	0.61397
1.5	0.01668	0.36492	0.61841
1.6	0.01661	0.36769	0.61570
1.7	0.01671	0.36679	0.61650
1.8	0.01662	0.36733	0.61606
1.9	0.01719	0.36708	0.61573
2.0	0.01631	0.36739	0.61630
2.1	0.01694	0.36721	0.61585
2.2	0.01687	0.36560	0.61753
2.3	0.01679	0.36932	0.61389
2.4	0.01671	0.36713	0.61616
2.5	0.01622	0.36185	0.62193
2.6	0.01643	0.36407	0.61950
2.7	0.01642	0.36457	0.61901
2.8	0.01613	0.36352	0.62035
2.9	0.01673	0.36689	0.61638
3.0	0.01658	0.37029	0.61313
3.1	0.01666	0.36929	0.61405
3.2	0.01638	0.36585	0.61777
3.3	0.01655	0.36601	0.61744
3.4	0.01632	0.36564	0.61804
3.5	0.01671	0.36710	0.61619
3.6	0.01653	0.36747	0.61600
3.7	0.01661	0.36793	0.61546
3.8	0.01618	0.36680	0.61702
3.9	0.01619	0.36279	0.62101
4.0	0.01553	0.36018	0.62429
4.1	0.01608	0.36287	0.62105
4.2	0.01657	0.36915	0.61428
4.3	0.01609	0.36456	0.61935
4.4	0.01643	0.36588	0.61769
4.5	0.01589	0.36543	0.61868
4.6	0.01697	0.37046	0.61257
4.7	0.01635	0.36421	0.61944
4.8	0.01679	0.36838	0.61483
4.9	0.01608	0.36347	0.62045
5.0	0.01644	0.36804	0.61552

Table S4: Prior probabilities, $\Pr((0,0)\,|\,x \not\leftrightarrow y), \Pr((1,0)\,|\,x \not\leftrightarrow y)$ for given population growth rate. $\Pr((1,1)\,|\,x \not\leftrightarrow y) = 0$ and $\Pr((0,1)\,|\,x \not\leftrightarrow y) = \Pr((1,0)\,|\,x \not\leftrightarrow y)$.

population growth rate	$\Pr((0,0)\,\|\,x \not\leftrightarrow y)$	$\Pr((1,0)\,\|\,x \not\leftrightarrow y)$
0.1	0.71860	0.14070
0.2	0.72326	0.13837
0.3	0.71825	0.14087
0.4	0.72074	0.13963
0.5	0.71840	0.14080
0.6	0.71535	0.14232
0.7	0.72101	0.13950
0.8	0.71763	0.14118
0.9	0.72282	0.13859
1.0	0.72071	0.13964
1.1	0.72033	0.13983
1.2	0.71690	0.14155
1.3	0.72133	0.13933
1.4	0.71767	0.14116
1.5	0.71695	0.14153
1.6	0.72500	0.13750
1.7	0.72197	0.13901
1.8	0.72570	0.13715
1.9	0.71812	0.14094
2.0	0.72632	0.13684
2.1	0.72068	0.13966
2.2	0.72049	0.13976
2.3	0.72274	0.13863
2.4	0.72434	0.13783
2.5	0.72076	0.13962
2.6	0.71979	0.14011
2.7	0.72178	0.13911
2.8	0.72418	0.13791
2.9	0.72191	0.13905
3.0	0.72564	0.13718
3.1	0.72764	0.13618
3.2	0.72513	0.13744
3.3	0.72460	0.13770
3.4	0.72546	0.13727
3.5	0.72482	0.13759
3.6	0.72594	0.13703
3.7	0.72528	0.13736
3.8	0.72663	0.13668
3.9	0.72640	0.13680
4.0	0.72666	0.13667
4.1	0.72441	0.13780
4.2	0.72865	0.13568
4.3	0.72687	0.13657
4.4	0.72509	0.13746
4.5	0.72841	0.13579
4.6	0.72341	0.13829
4.7	0.72125	0.13938
4.8	0.72730	0.13635
4.9	0.72314	0.13843
5.0	0.72477	0.13762
